# Nurse leaders’ experiences of remote leadership in health care

**DOI:** 10.1108/LHS-01-2023-0003

**Published:** 2023-05-08

**Authors:** Minna Hurmekoski, Arja Häggman-Laitila, Johanna Lammintakanen, Anja Terkamo-Moisio

**Affiliations:** Department of Nursing Science, University of Eastern Finland, Kuopio, Finland; Department of Nursing Science, University of Eastern Finland, Kuopio, Finland and City of Helsinki Social and Health Services, Helsinki, Finland; Department of Health and Social Management, University of Eastern Finland, Kuopio, Finland; Department of Nursing Science, University of Eastern Finland, Kuopio, Finland

**Keywords:** Remote leadership, Health care, Distributed organization, Nurse management, Nurse leadership

## Abstract

**Purpose:**

This study aimed to describe nurse leaders’ experiences of remote leadership in health care sector.

**Design/methodology/approach:**

Semistructured interviews were conducted among nurse leaders (*N* = 12) between January and March 2022. All of the interviewees had experiences of remote leadership and worked as immediate – (*n* = 5) or middle-level (*n* = 7) leaders in health care organizations across four provinces in Finland. The collected data were analyzed by inductive content analysis.

**Findings:**

The leaders had experienced a rapid transition to remote leadership and highlighted the need for guidelines and joint discussions with different stakeholders. The interviewees felt that working life has changed in the last two years and that remote leadership will now be a key part of leadership in health care. The leaders’ experiences highlighted how important trust is in remote leadership. Furthermore, the interviewees pointed out a need for face-to-face contact and described other good practices for remote leadership. Overseeing work-related well-being was also stressed as important in the remote context; however, the interviewees expressed a need for instructions and tools concerning the management of employee well-being. The sudden change to remote leadership was not only described as interesting but also challenging, which has affected the leaders’ work-related well-being. Support – both from the organization and other employees – was found to be crucial to health care leaders’ work-related well-being.

**Originality/value:**

The current study complements the little-researched topic of remote leadership in the health care sector. The results provide insights that can be used to develop remote leadership and/or guide future research.

## Introduction

Remote leadership has various definitions and has previously been described using several terms, such as digital leadership, e-leadership or e-HRM (Torre and Sarti, 2020; Terkamo-Moisio *et al.*, 2022; Klus and Müller, 2021). The common aspects of these definitions include a geographical and/or temporal distance between the leader and team members, as well as communication mainly occurring via information technology solutions (Cortellazzo *et al.*, 2019; Van Wart *et al.*, 2019; Tigre *et al.*, 2022). Furthermore, remote leadership may also describe a leader who employs digital solutions and electronic channels when managing their team and/or tasks or a leader who constantly interacts with technology (Cortellazzo *et al.*, 2019; Tigre *et al.*, 2022). In this study, the term remote leadership is used to describe situations in which a health care leader manages geographically-dispersed teams and uses information technology while consciously taking socioemotional aspects and organizations guidelines into consideration (Cowan, 2014). Thus, in this paper remote leadership is framed as an information technology-mediated, social process that changes individuals’ attitudes, behavior and engagement (Avolio *et al.*, 2001; Avolio *et al.*, 2014; Tigre *et al.*, 2022).

The development and widespread utilization of information technology have changed the way in which organizations function (Torre and Sarti, 2020) and how they are managed. Changes can be seen for example, in ways of communication, organizational patterns and management and leadership competences and practices (Van Wart *et al.*, 2019). At the same time, information technology has changed the way in which work is organized in sectors that have rigidly relied on the presence of employees, i.e. health care (Kiljunen *et al.*, 2022; Tigre *et al.*, 2022). In addition, the COVID-19 pandemic, including restrictions imposed by governments, have increased the share of remote leadership within the health care sector both in Finland and on a global level (Tigre *et al.*, 2022).

Prior to the COVID-19 pandemic, there was much of unrealized potential in digitalization of health care, for example, in the Europe. However, the implementation of digital health tools was hindered by individual concerns and organizational and systemic challenges (e.g. lack of legal framework) rather than by technical ones (WHO, 2021). Furthermore, the remote leadership in health care differs from various other sectors, as it is known to be human intensive area, in which most of the tasks are associated with various types of communication and presence requiring collaboration with patients or another professionals. Health care sector is also described as hierarchical and rigid context, in which remote working and remote leadership have been less scrutinized than for example in IT-sector (Kiljunen et *al.*, 2022). The COVID-19 pandemic accelerated the development of digitalization in health care sector globally, which has also led to changed expectations of patients/clients and health care professionals (WHO, 2021). More importantly, remote work and remote leadership became more common in health care during the pandemic, changing the understanding of work and leadership itself in health care.

Despite noticeable changes in how contemporary work is organized, previous scientific knowledge on remote leadership is limited and – in the worst case – fragmented (Cortellazzo *et al.*, 2019; Torre and Sarti, 2020; Tigre *et al.*, 2022), especially in health care (Terkamo-Moisio *et al.*, 2022; Kiljunen *et al.*, 2022). However, recent reforms and great investments in technology have led to a situation, in which distributed organizations with remote leadership practices are now commonplace within health care sector (Costa-Font and Turati, 2018; Kiljunen *et al.*, 2022). These changes have emphasized management of employee well-being as one of the main tasks in health care.

A new leadership paradigm has been predicted to emerge from the ongoing digital disruption. Traditional theories of leadership cannot adequately take the new context of remote work into account (Cortellazzo *et al.*, 2019; Torre and Sarti, 2020; Tigre *et al.*, 2022), which further highlights the need for empirical evidence from the health care sector (Terkamo-Moisio *et al.*, 2022; Kiljunen *et al.*, 2022). This article partly fills the existing knowledge gap by describing nurse leaders’ experiences of remote leadership.

### Perspectives on remote leadership

According to research from Tigre *et al.* (2022), remote leadership can be approached from four perspectives, namely, interpersonal orientation (e.g. successful interaction with others), personal attributes (e.g. management of one’s inner self), strategic focus (e.g. helping an organization achieve pre-determined goals) and delivery-related aspects (e.g. achieving the desired outcome). This highlights the wide range of leadership skills that are required from remote leaders (Maduka *et al.*, 2018; Laukka *et al.*, 2022). The mastery of these leadership skills has a positive impact on employees’ well-being (Chaudhary *et al.*, 2022). In addition, organizations should ensure the existence of standardized practices, which are important for successful collaboration between the remote leader and their teams; these practices and guidelines are also beneficial to setting achievable goals (Terkamo-Moisio *et al.*, 2022). Remote leaders can support employees in their work and strengthen their autonomy as well as their work-related well-being by setting clear goals, monitoring the development of certain skills and assessing whether set goals are achieved (Robert and You, 2018; Kilcullen *et al.*, 2021).

In the context of health care, a special feature of remote leadership is interactions between the leader and employees, with communication and trust being particularly relevant (Sinclair *et al.*, 2021; Terkamo-Moisio *et al.*, 2022; Kiljunen *et al.*, 2022), as they support work-related well-being, too. It has been stated that creating and enhancing the trust is more challenging than in the traditional face-to-face leadership (Turesky *et al.*, 2020; Terkamo-Moisio *et al.*, 2022). However, trust is essential for achieving the organization’s goals; therefore, remote leaders should actively take actions to build and enhance the interpersonal trust (Terkamo-Moisio *et al.*, 2022). Furthermore, the combination of an appropriate communication style and mutually created rules (e.g. concerning the regularity of communication) and practices (e.g. the used communication channels) are central for successful interaction in remote context (Cortellazzo *et al.*, 2019). Efficient communication is expected to be regular, open and unambiguous; this includes reciprocal, positive feedback that supports employees in the remote environment (Cortellazzo *et al.*, 2019; Sharpp *et al.*, 2019; Mutha and Srivastava, 2021; Terkamo-Moisio *et al.*, 2022). In addition to regular communication, trust is strengthened by a leader’s characteristics and leadership style, along with a psychologically safe environment (Norman *et al.*, 2020; Kilcullen *et al.*, 2021).

Both a leader’s digital skills (Liu *et al.*, 2018; Cortellazzo *et al.*, 2019) and technology-related challenges should be considered when studying remote leadership because the remote leader sets an example for how digital culture will be introduced to an organization (Sharpp *et al.*, 2019; Van Wart *et al.*, 2019; Kiljunen *et al.*, 2022). This highlights organizational support for remote leadership, for example, proactively introducing information technology solutions and setting standards, mentoring employees on how to use them and monitoring work performance (Sharpp *et al.*, 2019; Kilcullen *et al.*, 2021; Terkamo-Moisio *et al.*, 2022). However, remote leaders have described a lack of organizational understanding about the demands of remote leadership; thus, organizational support for leaders that focuses on the remote work context is crucial for the well-being and performance of leaders as well as their teams (Terkamo-Moisio *et al.*, 2022).

## Methods

### Aim

The aim of this study was to describe nurse leaders’ experiences of remote leadership in health care.

### Study design

Due to limited knowledge on the subject, a qualitative design was chosen to investigate participants’ perspectives of remote leadership and the meanings that they give to the phenomenon (Holloway and Galvin, 2016).

### Participants and recruitment

The study population consisted of nurse leaders who had experience of remote leadership. A convenience sampling approach was used (Polit and Beck, 2018). The researcher informed the participants about the study via e-mail. An appointment for an interview was made after a participant expressed their interest to participate.

A total of 12 female individuals who worked as immediate (*n* = 5) and middle-level (*n* = 7) leaders in health care organizations in four provinces across Finland were interviewed. All the leaders had at least two years of experience in remote leadership.

### Data collection

The data were collected in January–March 2022 through semistructured interviews (performed as both focus group (*n* = 3) and individual (*n* = 3) interviews). The interviews followed a previously designed interview guide that included questions about participants’ experiences of remote leadership, for example: “What kinds of remote leadership practices exist in you organization?” and “In your opinion, how did the COVID-19 pandemic influence the practices of remote leadership?”. The interviewees were asked additional questions when there was a need to gather more in-depth information on the topic. Apart from the participants’ name, no other background information was collected.

The interviews were carried out by two researchers (M.K and A.T.-M.) with the Microsoft Teams application (Microsoft Corporation, Redmond, WA). Both researchers participated in the interviews and agreed in advance about their roles. One of the researchers conducted the interview following the interview guide, whereas the other had more observing role. The interviews were recorded with the participants’ permission and oral informed consent was obtained from each participant prior to the interview and included in the recordings. The interviews lasted between 69 and 109 min, with an average duration of 99.5 min, which resulted in a total of 601 min of recorded material.

The material was transcribed verbatim by an external company (bound by confidentiality agreement). The transcription of the data yielded a total of 75 pages of text (font Calibri, 12 pt, spacing 1).

### Data analysis

Data analysis was carried out by two researchers (M.K and A.T.-M.) using inductive content analysis, as the previous knowledge of the phenomenon was limited, and the investigation focused on nurse leaders’ experiences (Holloway and Galvin, 2016; Polit and Beck, 2018).

First, the interview transcripts were read through several times to gain an overview. Next, sentences or a combination of phrases related to the research objective were marked as original expressions (*N* = 392). These original expressions were then reduced and grouped as subcategories based on similarities in the content. Thereafter, subcategories with similar contents were combined into upper categories and named based on their content. Finally, main categories were formed by grouping upper categories according to their content (Table 1). Both researchers (M.K. and A.T.-M.) analyzed the data independently and discussed the findings in several stages of the analysis process until a consensus was reached. In addition, the results of the analysis were discussed within the research group until a final consensus was achieved.

### Ethical considerations

The research followed the guidelines of the Finnish National Board on Research Integrity (TENK) and did not require ethical approval based on current Finnish legislation (TENK, 2019). Research permits were obtained from the participating organizations. Participants received a written information sheet that included information about the voluntary nature of participation, their right to withdraw from the study at any time and the possible risks and benefits of participation. Informed consent was obtained and recorded from each participant prior to the interview (TENK, 2019).

## Results

The data analysis yielded three main categories, namely, *remote leadership in changing working life, role of trust and reciprocal interaction in remote work* and *work-related well-being in the remote context.*

### Remote leadership in changing working life

This main category includes four upper categories, namely, *transition to complex remote leadership, need for guidelines and joint discussion, changing working life* and *perspectives on the future of leadership* (Table 2).

#### Transition to complex remote leadership.

Some of the interviewees described that remote leadership did – to some extent – exist prior to the COVID-19 pandemic; reasons for this were decentralized organizations and a large geographical area. However, the interviewees felt that the *transition to remote leadership* mainly started when COVID-19-related restrictions came into effect. They described the transition as a “high school of remote leadership” that improved digital abilities because they had to rapidly develop solutions for how to lead and remotely serve clients/patients.

The interviewees stated that the immediate superiors did not have sufficient time to lead as the COVID-19 pandemic and lack of nurses took a lot of energy; this led to a situation in which most of the day was spent providing guidance and/or organizing resources. The participating leaders shared negative experiences of this type of *fragmented leadership;* for example, one participant pointed out that they cannot “suppress fires all the time” and that they would require either double the time or a significantly smaller amount of personnel to appropriately lead their unit:

[…] the time is going to the organizing of resources, that one can’t lead well traditional, remotely or anyway. (I2P3)

The *tasks and role of the remote leader* were often mentioned in interviewees’ descriptions, a finding which highlights the implementation of a new leading culture that is based on trust and collaboration. The interviewees also pointed out that a leader should delegate tasks, which would help the employees independently come up with solutions to problems, as the leader is not needed for every task or problem. The participants felt that leaders should work to enable employees and highlighted that the time of continuous control is over. They also felt that autonomy leads to better results than control. The interviewees also described the remote leader’s role as supportive, showing respect and trust to employees, increasing their commitment. In addition, the leader should be aware of how changes in patient needs can affect services and that they can rely on employees’ expertise when necessary. According to the interviewees, the remote leader should be accessible when needed, as well as support and enable interactions within the unit. This was expressed in the interviews as follows:

[…] the task of the leader is to be an enabler, so that he/she creates possibilities to do that work and lead the expertise […] so they should adopt the perspective that a leader offers support whenever it is needed. (I1P3)

The interviewees described reduced costs and saved working time as *benefits of remote leadership*. Furthermore, they felt that meetings with many participants were more efficient in the remote context. Some of the interviewees pointed out that making changes or decisions within a large group is easier in the remote than face-to-face setting as they receive knowledge and support for their work. However, the interviewees expressed some *ethical concerns in remote leadership* that were related to data security, professional confidentiality and employee equality:

[…] maybe what is highlighted in this remote context is exactly that, what kind of information can we transfer by which channel so that no patient/client information is jeopardized. (I6P2)

#### Need for guidelines and joint discussion.

The interviewees described the *lack and inadequacy of guidelines* regarding remote leadership, which they partly associated with the speed of the transition to remote work and the extraordinary situation of the COVID-19 pandemic. Some of them shared that they had received oral instructions, which mainly addressed restrictions on assembly and distribution of information via virtual channels. This was described as the current situation at the time of the interviews, with one participant stating:

We probably don’t have any guidelines for remote leadership, like something official, written, […] At least I don’t perceive so. (I2P1)

The lack of guidelines led to the *creation of own practices,* which were largely based on the leaders’ previous experiences and professional skills. The interviewees described that they “somehow” started to remotely lead and applied different approaches based on the situation. The chosen type of leadership was based on considerations about how to act and which approach is appropriate to which person. The interviewees pointed out that the existing administrative structures at organizations were – to a large part – brought to the virtual context. This way the employees knew that they could rely on the unchanged administrative structures:

The [remote]leadership was kind of shaped by what type of situations one has been in, and then this is applied to how one leads. So, this is how it [remote leadership] has gone so far. (I4P1)

The interviewees expressed a *need for joint discussion* between different stakeholders and organizations about remote leadership. They pointed out that assessing the current situation and the experiences of the past years would enable them to evaluate how remote leadership is working as well as recognize the benefits, disadvantages, possibilities and risks. Furthermore, they highlighted that knowledge sharing between different organizations could give ideas for the development of remote leadership. The interviewees described a need for congruent practices, as well as protocols that ensure the consistency of remote leadership. These were seen as a means to avoid arbitrariness and unit-specific practices, which can negatively affect work-related well-being, among other aspects. One interviewee shared:

[…] we need to create certain protocols, so that remote leadership will be carried out in a similar fashion, not that there will be arbitrary developments or certain unit-specific practices […]. (I1P3)

#### Changing working life.

The interviewees talked in length about *increasingly common remote work,* stating that nearly all of them worked remotely during the pandemic time. The interviewed leaders described how they had made agreements between one another and their employees about the amount and times of remote work. These agreements were partly instructed from the organization. Some of them shared that they had made remote work agreements with all of their employees, whereas others described discussions with their employees in which they had to justify why remote work is not possible in their units. The interviewed leaders highlighted that the possibilities for remote work are based on the nature of work itself and, therefore, the strict governmental rules are not necessarily applicable. Based on the descriptions, it was clear that hybrid working, in which work is performed remotely to some extent, has become the most common way of working in most of units:

Sometimes we were completely remote, after which there has been the possibility to work in the unit, although remote work was recommended […] at the moment, we have this hybrid model which, hopefully – also from my own point of view – remains. (I6P2)

The interviewed leaders pointed out that *work-related attitudes* have changed during the COVID-19 pandemic. They felt that employee attitudes have become more modern, including more positive views of remote work and the associated productivity. The predominant opinion among the interviewees was that the way of working has moved forward, and they also described the presence of attitudes questioning whether the status quo prior to remote work was old-fashioned. However, some of the interviewees highlighted the traditional understanding of employment and ways of working that still prevail in health care. They further pointed out that the change in the mode of working has been so big that it occasionally leads to negative feelings:

I didn’t feel that way previously, that there would not be trust [in remote work], but it has somehow increased, as everybody is working remotely, so the attitude towards remote work has become quite different. (I6P1)

When asked about *the development of information technology and competences,* the interviewees talked about functioning systems, applications and devices. They felt that their organization offered a good Internet connection and that the IT departments reacted rapidly to the change to remote work. Furthermore, according to the interviewees, employees had a wide range of competences in information technology solutions, although the level of competence may have varied. However, the leaders described the rapid improvement of these competences, which was aided by peer and organizational support. One interviewee stated:

[…] and also, as we started working remotely on a fast schedule, the IT department reacted rapidly, so we had access to certain networks and places, so that came fast to the leaders, and so as we got started, things started to go well. (I6P2)

The interviewees also mentioned *employees’ autonomy and self-direction*. They discussed how the transition to remote leadership has been easier for teams that were used to making independent decisions and working autonomously. Furthermore, the interviewees highlighted how positive team culture and good intrateam relationships benefit autonomous working. An employee’s self-direction was seen as the ability to make independent decisions, self-confidence and responsibility for their work. The leaders pointed out that employees should report possible deficiencies in their skills and request training, as this is difficult for leaders to assess:

[…] when the intrateam relationship is good, and there is different kind of expertise then the team is able to work efficiently and solve problems. (I5P1)

#### Perspectives on the future of leadership.

Some of the interviewees found it *difficult to assess* the future of leadership in health care. Due to the scope of change, they felt that it would be difficult to predict how it will further progress. The interviewees also expressed worries about what is coming next and what kinds of systems they will still need to learn how to use. In addition, they felt that the stabilization of the current activity demands a lot of work. Some of the leaders also expressed critical opinions about the suitability of remote leadership to the health care context. For instance, one leader shared:

Then I am a bit afraid of what is coming next, which is better than this [name of application] and we need to learn some new system. So I have this fear, when my own head starts to get a little old, that do I still need to learn something? (I2P1)

On the other hand, several interviewees stated that there is *no turning back* to traditional, face-to-face leadership. In their perspective, the increasing digitalization and reform of health care has created a situation in which work is increasingly done regardless of time or place. Furthermore, they stated that remote leadership has too many advantages to be given up. They further supported this point of view by listing the experiences and competences they had gained and stated that remote leadership should even be broadened from the current scope:

There is no turning back to the old way after this, like, first of all this different “digiworld” will affect us in the way that work will be more often done regardless of place and time via high-quality remote connections. (I1P3)

The interviewees also expressed an *expectation of hybrid leadership* in terms of certain face-to-face contact with employees at specific times. They further stated that employees will not be satisfied with the traditional way of working and that the hybrid model would bring flexibility to their work. A leader’s improved ability to interpret the situation within the unit was highlighted as one benefit of hybrid leadership, with one leader specifying:

[…] the hybrid model will be the reality, the employees will no longer agree to doing things in the way of the old world now that we have learned this hybrid approach, so the hybrid way will probably be the commonplace practice and it brings that kind of flexibility of work that employees want. (I1P3)

### Role of trust and reciprocal interaction in remote work

Aspects related to trust, interaction and communication were presented by the upper categories *trust and its consequences* as well as *interaction and communication in the remote context* (Table 2).

#### Trust and its consequences.

According to the interviewees, *trust between the leader and employee* is essential in the remote context. The interviewees stated that clinical work is carried out independently; as such, employees require their leader’s trust to be successful. The interviewees felt that leaders demonstrate trust to employees by avoiding unnecessary control, informing them about upcoming issues and being accessible despite remote collaboration. Moreover, the interviewees felt that leaders should keep their promises and support the organization to enhance trust. An improvement in results was seen as a *consequence of trust*. Employees who trusted their leader contacted her/him less frequently, as detailed by one interviewee:

[…] somehow I think that we have a lot of things that are done independently in the field, and it doesn’t work if we don’t trust employees to do their job. (I3P1)

#### Interaction and communication.

The *need for face-to-face contact* was highlighted in the interviews. The interviewees stated that the employees miss the presence of their leader, as well as consider face-to-face contact to be most fruitful because it enables the informal communication that is necessary in certain situations, for example, a difficult life situation. The interviewees described a new kind of appreciation toward colleagues that had begun during the pandemic. Face-to-face contact was regarded to be especially important to get to know new employees, planning activities and a leader’s observation of issues in the team that are not directly related to the work:

That has – in my opinion – changed after that [pandemic], as if it was a kind of force to work remotely […] like maybe we learned to appreciate that one is allowed to come to work in the morning and be with the colleagues. (I3P1)

The interviewees regarded multitasking as one of the *challenges of remote communication*; for example, they described situations during which a colleague was doing something else during the meeting and not actively taking part in the discussion until they were asked a direct question. However, they felt that with time people have gotten to understood that multitasking does not function during virtual meetings. Another challenge was related to differences between individuals; notably, someone who was shy stood out during virtual meetings, e.g. being silent or not opening the camera. The interviewees also shared that technical difficulties and the lack of competence in this area led to challenges in remote communication. They also felt that certain types of communication are missing from the remote context; this means that there is a risk of misunderstanding, as moments of silence are easily filled with assumptions. In addition to misunderstandings, the interviewees stated that remote communication increases the risk of conflicts within the team, and that the remote setting decreases a leader’s ability to observe these possible conflicts. For example, one leader shared the following:

People are doing several things or focusing on other issues, as they are listening to the meeting. They are quiet and do not comment, unless one poses a direct question. (I2P2)

Organizing regular meetings was one of *the good practices* described by the interviewees. Some of the interviewees stated that there was no agenda for the meetings, which meant that they were able to discuss issues that had been raised during the week. In addition to these meetings, leaders highlighted the regular distribution of information via e-mail. They found the clear route through which information travels from the top level of management to the unit level to be helpful. The interviewees felt that the use of the camera in virtual meetings improved nonverbal communication. The interviewees also mentioned rules that they had created together with their employees, such as adhering to the agreed schedule(s) and preparedness when joining meetings. To enhance accessibility, the interviewees detailed the use of open calendars so that the weekly plan was visible for everyone, with one leader sharing:

[…] I have everything in [name of the application] calendar, I have put there all the things that I do, because I also plan my work week, workdays like this, but then it is also visible to my own superior. (I6P2)

### Work-related well-being in the remote context

The two upper categories of this main category were based on the interviewees’ descriptions of work-related well-being, namely, *leading work-related well-being* and *aspects associated with work-related well-being* (Table 2).

#### Leading work-related well-being.

The interviewees stated that they have *set the focus on work-related well-being* in the remote context by discussing how their employees are coping at work with other leaders. According to the interviewed leaders, work-related well-being is also a theme in the goal and development dialogues that the leaders have had with their employees. The interviewees pointed out that the focus should also include the work-related well-being of the leaders. However, the interviewees touched upon the lack of *tools to lead work-related well-being* by stating that they have very little concrete instructions or means to oversee work-related well-being when in a remote position. For example, one of the leaders detailed:

There has been a lot of discussion about this [work-related well-being], but very little, in my opinion, concrete instructions concerning how we could take action. (I6P2)

#### Aspects associated with work-related well-being.

The interviewees described the *interesting speed of change* related to the use of information technology solutions and new ways of working during the pandemic. The change was described as challenging, yet the interviewees found it interesting and felt that they have coped with the changes as they had no other choice:

My head got a bit dizzy when you think that it has been such a short time, and we fluently use all kinds of means, as we were kind of forced to learn them. A human learns strange things when presented with no other choice. (I2P1)

The changes which had occurred were associated with the *load of the leadership,* with the interviewees sharing that this negatively influenced work-related well-being. The interviewees also felt that there is a lack of personnel for sufficient patient care. They felt pressure, responsibility and the threat of someone getting sick. Furthermore, they described the *loneliness of remote work* and expressed how it can significantly influence team commitment and the mental burden of employees. In addition, they stated that – for remote workers – the border between working time and private life becomes hazy, which can lead to *over-efficiency of work;* the leaders mentioned that – in some cases – they had to intervene to protect an employee’s well-being. Another well-being concern of remote work was *ergonomics.* An interviewee discussed the association between remote work and work-related well-being as follows:

[…] for some employees we had to occasionally take away their work equipment, as it [working time] has faded and they have started to work all the time, so that they have had problems with coping with work […] (I6P3)

The interviewees felt that the *flexibility of work* positively influences work-related well-being as it enables participation in, for example, networking or education. The interviewees also noted that combining work with family life has become easier as the individual life situation may be taken into account. They also felt that they have gained distance and peace in work through remote or hybrid work. However, the interviewees did highlight the significance of *complex, reciprocal support* to well-being in the sense that it should always be available. The interviewees felt that they supported their employees but received very little support themselves, with one interviewee discussing the need for support from the organization and other employees as follows:

[…] and I always see the leadership as bidirectional, also the role of employees in supporting the leader and how it can be realized in this kind of world. (I4P1)

## Discussion

This study contributes to the limited (Terkamo-Moisio *et al.*, 2022; Kiljunen *et al.*, 2022) scientific knowledge base concerning remote leadership in the health care sector by describing Finnish nurse leaders’ experiences. The results provide insight into the development and current state of remote leadership in the health care sector, which is important for a topic that has been previously labeled as fragmented (Cortellazzo *et al.*, 2019; Torre and Sarti, 2020; Tigre *et al.*, 2022). Furthermore, the results highlight aspects of remote leadership that should be further developed and focused on when educating future leaders.

It is noteworthy that the interviewees in this study mainly approached remote leadership from an interpersonal perspective, e.g. communication and the leader–employee relationship. In addition, the participants approached remote leadership through different stakeholder abilities or personal traits, like the remote leader’s tasks and role. This way of thinking is in line with what was previously reported by Tigre *et al.* (2022). Communication and interpersonal relationships have been found to be the most studied aspects of remote leadership in health care (Cortellazzo *et al.*, 2019; Kiljunen *et al.*, 2022). Future research should pay more attention to the strategic focus and delivery-related aspects of remote leadership (Tigre *et al.*, 2022) to provide a more comprehensive overview of the phenomenon.

The interviewees highlighted the lack of organizational guidelines and practices for remote leadership aimed to the unified way of leadership within the organization, for example, addressing the ways and amount of remote work or communication. The lack of the guidelines led to situations where leaders had to quickly develop individual practices based on their own expertise. This, in addition to the fact that these practices were developed and reformed by nurse leaders on a day-to-day basis, raises concerns about the coherence of remote leadership within health care organizations. Furthermore, at the time of the interviews, the participants had at least two years of experience in remote leadership due to the COVID-19 pandemic, but the lack of guidelines was still apparent in their descriptions. This result emphasizes the significance of organizational guidelines and joint discussions, as pointed out by the interviewed nurse leaders as well as previous literature (Turesky *et al.*, 2020; Terkamo-Moisio *et al.*, 2022). Hence, the results stress that organizations should strive to develop uniform practices and guidelines based on the scientific literature and existing experiences of remote leaders.

The results show that remote leadership has been practiced prior to the COVID-19 pandemic but was not as evident. This is in line with the research of Torre and Sarti (2020), who stated that even when remote leadership was carried out, it was never been officially recognized by top-level management. The views detailed in the present study further support the suggestion that a new paradigm of remote leadership is emerging, as has been chronicled in prior research (Cortellazzo *et al.*, 2019; Torre and Sarti, 2020; Tigre *et al.*, 2022). Also, the interviewees of the present study clearly stated that remote leaders should understand that the time of control is over; instead, a leader’s task is to implement a new culture based on trust and collaboration, which may require certain leaders and organizations to reconsider the concept of remote leadership in health care.

The leaders who participated in the current study felt that remote leadership will also have a central role in the future of health care. The need of face-to-face meetings and presence that emerged in our results may be a consequence of the traditional understanding of leadership in health care. However, it should be taken into account in developing and redefining the different forms of leadership in the health care sector, as the professionals’ and patients’ expectations regarding the work and health care services have changed in the past years. This issue remains – to the best of our knowledge – understudied and should be considered in future research. The presented results answered another understudied aspect of remote leadership (Kiljunen *et al.*, 2022), as the interviewees mentioned that they saved both time and money through remote meetings. However, more robust research is needed to show possible effects and efficiency of remote leadership.

The essential role of trust and communication was evident in the results of current study and reflected what has been reported in previous literature (Cortellazzo *et al.*, 2019; Sinclair *et al.*, 2021; Kiljunen *et al.*, 2022; Tigre *et al.*, 2022*)*. This finding may be partly attributed to the interactive nature of the health-care profession (Terkamo-Moisio *et al.*, 2022). The presented good practices included regular meetings and information, turning on the camera in virtual meetings to enhance non-verbal communication and the creation of common rules. These practices were also found to be beneficial in previous literature (Cortellazzo *et al.*, 2019; Terkamo-Moisio *et al.*, 2022; Kiljunen *et al.*, 2022) and should be considered when developing education for future leaders. In addition, current nurse leaders should be offered the possibility to enhance their skills related to communication. Furthermore, organizations should take actions, such as mentoring and support, that progress the development of an open and safe work environment (Sharpp *et al.*, 2019; Terkamo-Moisio *et al.*, 2022).

Unexpectedly aspects of work-related well-being emerged as a result of inductive analysis revealing its multifaceted nature. This is critical for organizations to consider, as work-related well-being has been associated with nurse leaders’ turnover intentions and burnout (Niinihuhta *et al.*, 2022). Moreover, the balance between personal and work life, overall workload and role strain have all been associated with nurse leaders’ work-related well-being and – as an extension – their commitment to the organization and ability to cope (Niinihuhta *et al.*, 2022). Furthermore, leaders’ mastery of core e-competences (e-communication skills, e-change management skills and e-technology skills) has a positive impact on employees’ work-related well-being (Chaudhary *et al.*, 2022). The current results highlight how remote leaders will require additional support to overcome the stress associated with remote work; this issue has also been covered in previous literature (Terkamo-Moisio *et al.*, 2022). This perspective should be included in future studies of remote leadership if organizations are to have a solid basis from which to sufficiently develop remote leadership.

### Strengths and limitations

To enhance credibility, the research process has been described in detail. It further strengthens the dependability of the study and enables replicability in other contexts (Polit and Beck, 2018). Original excerpts were presented in the results to demonstrate the connection between the results and the original data. Even though data saturation was achieved, the sample size was moderate; therefore, the results may not be directly transferrable or generalizable to other contexts. Furthermore, the fact that all of the participants were women may have influenced the results, even if the gender distribution closely mirrored the gender distribution among health care personnel, where 90% of health-care leaders are female. All of the participants had experiences of remote leadership, which strengthens the reliability study. The results are presented and discussed objectively to strengthen the confirmability of the study.

## Conclusions

The transition to remote leadership mainly occurred at the start of the pandemic due to government restrictions, but the experienced benefits, along with changes in how work is perceived, mean that remote leadership will have a key role in future health care. Hence, there is a need to update leadership education by including certain key aspects of remote leadership, i.e. communication and trust building. Furthermore, multiform education and virtual group works would enhance the leadership students to develop their leadership skills in remote context as well as strengthen their digital abilities. Current leaders should also be offered possibilities to enhance their skills so that they can lead their remote teams more effectively. Organizations should create uniform practices and guidelines for remote leadership, as there is currently a clear lack of these. Furthermore, it would be important to use leaders’ existing experience and scientific knowledge when developing and improving remote practices at health care organizations. The present study has provided certain insight into how leaders experience remote leadership, yet more research in this area is needed due to the lack of scientific knowledge, especially research that adopts a strategic focus and considers delivery-related aspects of remote leadership.

## Figures and Tables

**Table 1.**
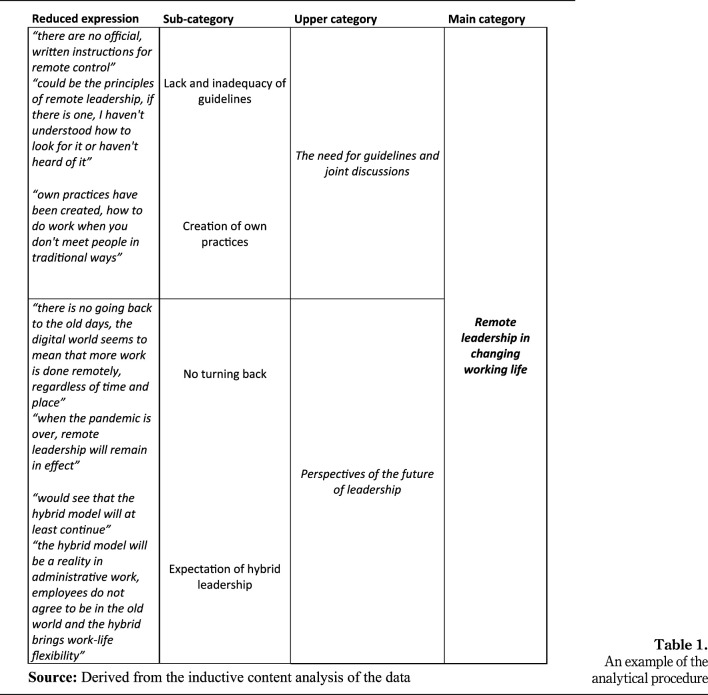

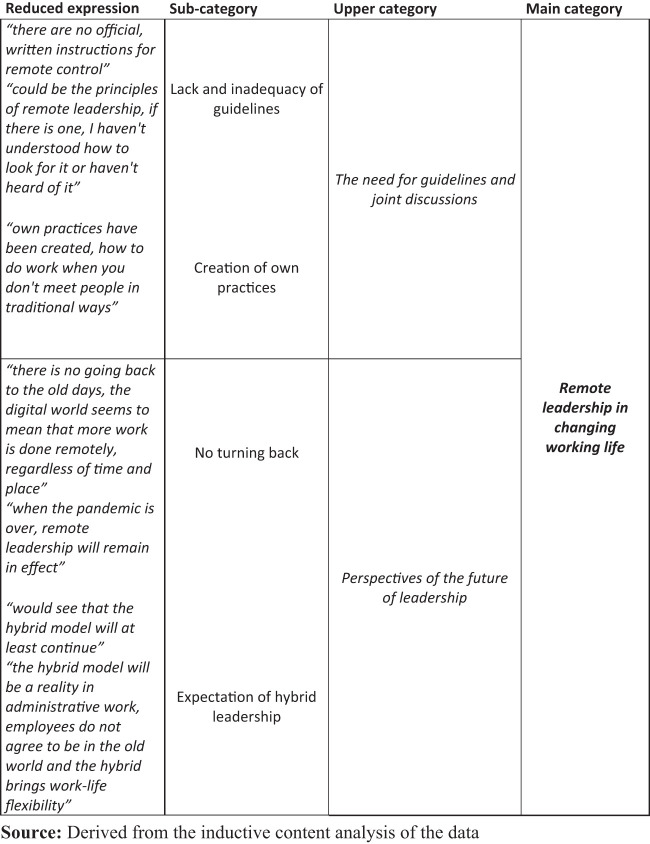
An example of the analytical procedure

**Table 2.**
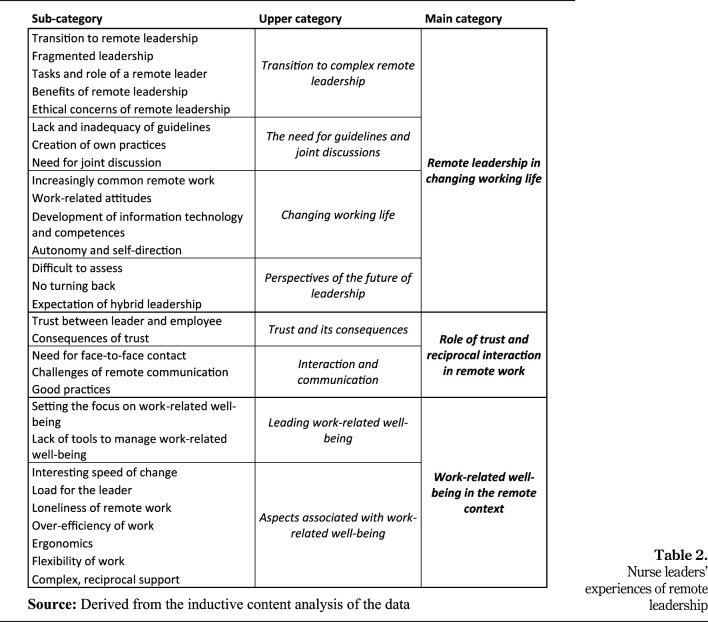

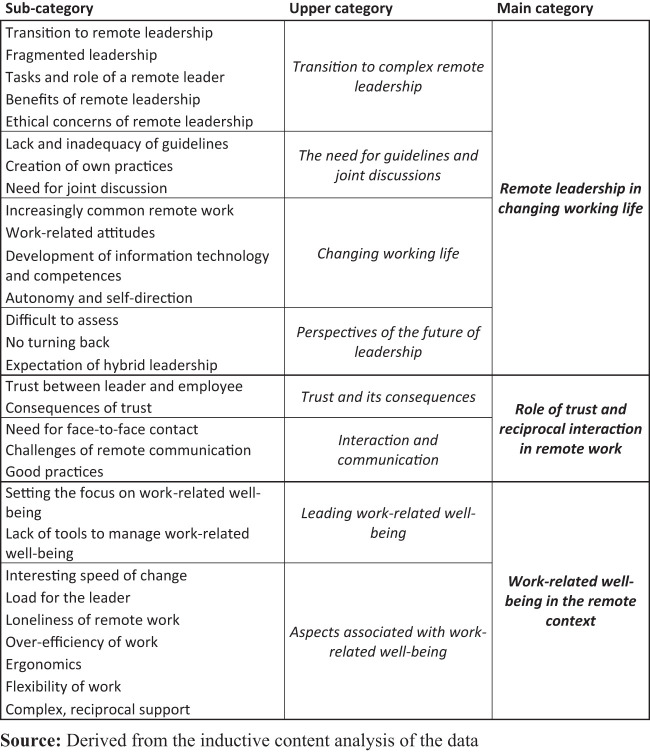
Nurse leaders’ experiences of remote leadership
